# Overview of extracellular vesicle characterization techniques and introduction to combined reflectance and fluorescence confocal microscopy to distinguish extracellular vesicle subpopulations

**DOI:** 10.1117/1.NPh.9.2.021903

**Published:** 2022-04-04

**Authors:** Canan Bağcı, Melike Sever-Bahcekapili, Nevin Belder, Adam P. S. Bennett, Şefik Evren Erdener, Turgay Dalkara

**Affiliations:** a Hacettepe University, Institute of Neurological Sciences and Psychiatry, Ankara, Turkey; b Bahçeşehir University, Department of Biomedical Engineering, İstanbul, Turkey; c Ankara University, Institute of Biotechnology, Ankara, Turkey

**Keywords:** extracellular vesicles, confocal microscopy, imaging, reflectance, fluorescence, brain

## Abstract

Extracellular vesicles (EVs) are nanoparticles (30 to 1000 nm in diameter) surrounded by a lipid-bilayer which carry bioactive molecules between local and distal cells and participate in intercellular communication. Because of their small size and heterogenous nature they are challenging to characterize. Here, we discuss commonly used techniques that have been employed to yield information about EV size, concentration, mechanical properties, and protein content. These include dynamic light scattering, nanoparticle tracking analysis, flow cytometry, transmission electron microscopy, atomic force microscopy, western blotting, and optical methods including super-resolution microscopy. We also introduce an innovative technique for EV characterization which involves immobilizing EVs on a microscope slide before staining them with antibodies targeting EV proteins, then using the reflectance mode on a confocal microscope to locate the EV plane. By then switching to the microscope’s fluorescence mode, immunostained EVs bearing specific proteins can be identified and the heterogeneity of an EV preparation can be determined. This approach does not require specialist equipment beyond the confocal microscopes that are available in many cell biology laboratories, and because of this, it could become a complementary approach alongside the aforementioned techniques to identify molecular heterogeneity in an EV preparation before subsequent analysis requiring specialist apparatus.

## 1 Introduction

Extracellular vesicles (EVs) originate from membranes of all cell types and can be found in all tissues and biological fluids.^1^^–^^4^ They are ∼30 to 1000 nm in diameter and play a crucial role in intercellular communication by delivering a variety of cargo, including nucleic acids, proteins, glycans, and lipids to local and distant cells where they influence cellular phenotype.^2^^,^^3^^,^^5^^,^^6^ EV-mediated intercellular signaling contributes to the regulation of a wide range of biological functions in target cells including immunological processes (e.g., antigen presentation), communication between components of the neurovascular unit, glial and neuronal function, development and differentiation of stem cells, and tissue regeneration. Additionally, in aberrant conditions, EVs have been associated with the pathophysiology of stroke, Alzheimer’s disease, Parkinson’s disease, cancer, obesity, cardiovascular disease, and rheumatoid arthritis.^2^^,^^5^^,^^7^^–^^11^ Therefore, there is a rapidly increasing scientific interest in EVs including their detection and imaging in tissue or biological fluids.

EV membranes are rich in lipids, such as sphingomyelin, phosphatidylserine, cholesterol, and ceramides. While EVs are heterogenous, they can be categorized as exosomes or microvesicles depending on their subcellular origin. Exosomes (<150  nm in diameter) have an intracellular origin, forming through invagination of endosomal membranes before being released upon fusion of the multivesicular endosome with the plasma membrane. In contrast, microvesicles (also called ectosomes) are formed by outward budding of the plasma membrane and range from 50 to 1000 nm in diameter. Because exosomes and microvesicles cannot be distinguished by size alone, and their distinction is also complicated by overlapping densities of constituent proteins, the term EVs is used to describe these populations unless their subcellular origin can be verified.^12^ Different subpopulations of EVs are generated depending on their mode of biogenesis which can be driven by sphingomyelinases, tetraspanins, lipid translocation enzymes, and components of the endosome sorting complex required for transport (ESCRT) that induce membrane curvature. These biogenesis pathways determine a vesicle’s molecular composition and shared molecular pathways across cell types result in EV populations with common proteins, such as tetraspanins {cluster of differentiation [Cluster of differentiation (CD)] 9, CD63, and CD81}, ESCRT-associated proteins (ALIX, TSG101, and syntenin), integrins, heat shock proteins (HSP70 and HSP90), actin, tubulin, and flotillins.^13^^–^^18^ The EV content also exposes the interior of cells from which they are secreted. EVs can be released by all cell types, including endothelia, pericytes, vascular smooth muscle cells, as well as central nervous system (CNS) parenchymal cells, and are found in biological fluids such as cerebrospinal fluid (CSF), plasma, serum, semen, saliva, and urine.^19^ Since surface markers on EVs reflect their parental cell origin, these proteins can be utilized for selective isolation and identification of cell type specific EVs. Neuronal L1 cell adhesion molecule (L1CAM) is an example of a specific surface protein marker used to capture neuron-derived EVs from brain tissue and CSF,^2^^,^^14^ although its usage outside the CNS is limited because of its expression in non-neuronal cell types and the presence of soluble L1CAM in the blood.^20^ Aldehyde dehydrogenase 1 family member L1 (ALDH1L1), a marker of astrocytes, was shown to be found in EVs derived from CSF samples of patients suggesting that it can be used for the identification of astrocyte derived EVs.^21^

The EVs in a biological sample are heterogenous. This arises because a single cell type produces different populations of EVs from distinct subcellular locations; various EV biogenesis pathways may operate in those locations giving rise to, e.g., multiple subpopulations of exosomes released from one multivesicular body; the EVs in a biological sample originate from numerous different cell types; and the physiological conditions experienced by a particular cell alter the composition of secreted EVs.^22^ This heterogeneity, together with the small size of EVs, complicates their analysis.^23^ Several techniques are available to characterize their size, concentration, and composition, such as flow cytometry (FC), resistive pulse sensing, nanoparticle tracking analysis (NTA), and small-angle x-ray scattering.^24^ Optical methods have also been employed to assess the size, concentration, morphology, biochemical composition, and cellular origin of single EVs.^25^^–^^27^ In this paper, we will discuss available methodologies for their detection and characterization, especially their imaging with conventional confocal microscopy as a bench-side practical approach.

## 2 Extracellular Vesicle Characterization Methods

Many methods exist to enrich EVs from biological samples, and these include ultracentrifugation, ultrafiltration, immunoprecipitation, and microfluidic technologies,^28^ as well as commercially available kits.^29^ Although EVs can be efficiently isolated with any of these methods, there are no rapid and standardized methods for characterization of their cellular origin, which is essential for utilizing EVs to gain insight to the physiological processes and pathophysiology of diseases at the cellular level.^30^ For example, selective isolation of EVs derived from the component cells of the neurovascular unit (e.g., endothelium, pericytes, and astrocyte endfeet) from the peripheral blood could provide a window to simultaneously investigate several biological processes in these cells *in vivo* and over time. The isolated EVs could then be characterized with methods outlined below and visualized either by light scattering (due to their refractive index being different to their surrounding aqueous medium^25^), or by specifically tagging the EVs with fluorophores or quantum dots.^31^

### 2.1 Dynamic Light Scattering

A technique based on the similar imaging principle is dynamic light scattering (DLS), which is also known as photon correlation spectroscopy. Similar to NTA, it relies on a monochromatic coherent laser beam passing through the particle suspension to calculate the particle velocity distribution caused by Brownian motion,^24^ then yields the particle size, and density by analyzing the fluctuations in scattered light using Stokes–Einstein equation. ^32^^,^^33^ However, DLS uses photon detectors instead of a camera; therefore, it does not directly visualize the EVs but evaluates the entire sample and yields a distribution plot.

Although DLS is a rapid and highly sensitive method with a measurement range from 1 nm to 6  μm, which does not require a pre-treatment process, the data obtained from DLS analysis are only reliable for monodisperse suspensions. In polydisperse suspensions, the light scattered from larger particles obscures that from small particles.^34^^,^^35^ Hence, analysis of heterogeneous EV populations is limited with this technique.

### 2.2 Nanoparticle Tracking Analysis

**Fig. 1:**
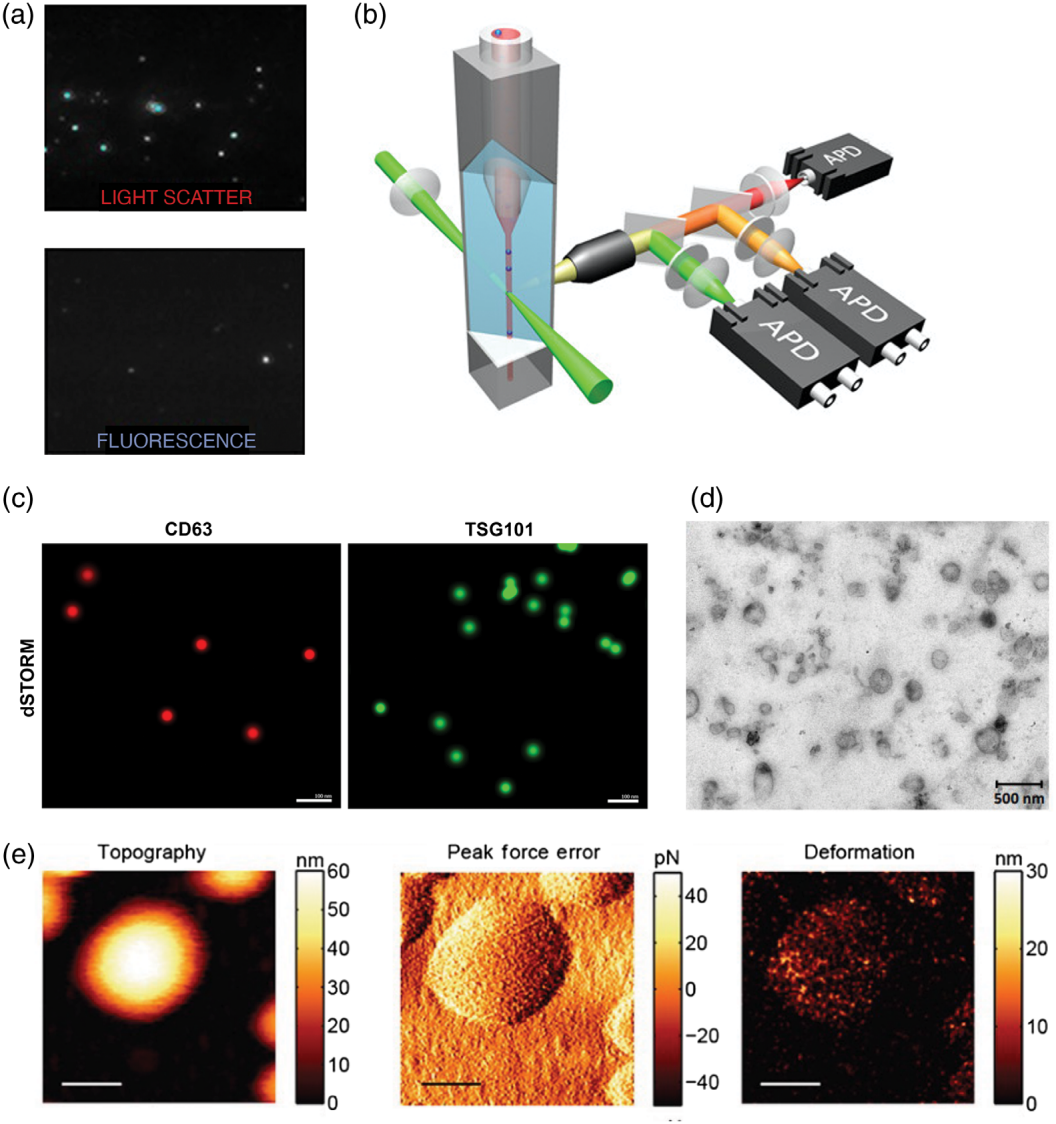
(a) NTA of canine placental mesenchymal stem cell EVs in light scatter mode (top panel), and fluorescence NTA of the same samples labeled with quantum dot-bound antibodies targeting CD9 (bottom panel).^37^ (b) A schematic depicting a nFC, where single EVs are passed in suspension through a laser beam to generate photons which are detected by three single-photon counting APD, enabling multiparameter detection of two-color fluorescence and side-scattering of EVs. (c) dSTORM super-resolution images of EVs derived from human glioblastoma cells stained with antibodies targeting CD63 (red) and TSG101 (green).^41^ (d) Polydisperse EVs released by the helminth pathogen, *Fasciola hepatica*, imaged by TEM.^42^ (e) AFM used to define the size and shape of EVs and showing the deformation on the EV surface after the application of increased force through the cantilever.^43^ (c) and (e) Scale bars: 100 nm and (d) 500 nm. (a) and (c)–(e) Reproduced with permission, under Creative Commons BY 4.0 license. (b) Adapted with permission from Ref. 44. Copyright 2018, American Chemical Society.

Nanoparticle tracking analysis (NTA) is one of the most commonly used biophysical techniques for characterizing EVs by size and quantifying EVs. NTA can determine the size and concentration of EVs in solution prepared from a wide variety of samples.^36^^–^^39^ This technique monitors the Brownian movement of particles in liquid suspension in real time and can estimate EV sizes ranging from 60 to 1000 nm.^40^ Brownian motion of the particles is measured using laser light scattering microscopy, with a camera as the detector, and their hydrodynamic diameter is calculated using the Stokes–Einstein equation. This can yield the particle concentration and size distribution in the sample, although NTA alone is unable to distinguish EVs from other particles that confounds measurements of EV preparations. To enhance NTA specificity, fluorescence NTA can be performed where EV proteins are stained with antibodies conjugated to fluorophores [Fig. 1(a)] and then only fluorescently labeled particles are detected and characterized within the solution.^45^ However, this approach can be limited by bleaching of the fluorophore, and while labeling EVs with antibodies conjugated to quantum dots can enhance photostability, this restricts the targets against which antibodies can be selected and additional purification steps are required to separate the EVs from unbound fluorophores.^37^

The advantages of NTA are that a relatively short time is needed for sample preparation and measurements (<1  h) and the EVs can be analyzed in their native form in solution. However, this technique biases the detection of larger particles in a solution since the intensity of the scattered light corresponds to the sixth power of the diameter of the particles. Accordingly, small particles in solutions may be underrepresented and an optimal dilution should be used during sample preparation such that masking of smaller particles by larger particles can be reduced and NTA camera is able to detect all particles in the specimen.

### 2.3 Flow Cytometry

Flow cytometry (FC) enables detection and characterization of the cytoplasmic or surface proteins of EVs.^19^^,^^46^^,^^47^ Conventional FC allows the measurement of relatively large-sized EVs (≥300  nm).^47^ In FC, a laser beam of a specific wavelength is directed at a hydrodynamically focused stream of fluid containing suspended particles [Fig. 1(b)].^48^^–^^50^ At the point where the stream of fluid passes through the laser beam, a number of visible and fluorescent light detectors are present.^47^^,^^51^^,^^52^ One of these detectors is placed in line with the light beam and measures the forward-scattered light (FSC). Another detector placed perpendicularly to the stream is used to measure the side-scattered light (SSC). In general, FSC assesses physical characteristics such as the relative size, whereas SSC reflects the inner complexity of the particles such as granularity.^26^ Conventional FC is unable to detect particles that have a diameter <300  nm due to their limited sensitivity and resolution, leaving small EVs outside the detection limits.^28^^,^^47^^,^^49^^,^^53^ To improve the sensitivity of conventional flow cytometers, several alternative solutions have been reported. For instance, small EVs can be detected by conjugating them to micrometer-sized latex beads with specific antibodies against antigens found on the EV membrane surface.^26^^,^^49^ More recently, FC instruments with enhanced detectors, lower electronic noise, optimized laser excitation, laser beam shaping, and more specific probes have been developed, which can detect particles smaller than 300 nm. Analysis algorithms can also help distinguishing EVs from aggregates and noise.^49^^,^^53^ Another notable development in instrumentation used for FC is nanoscale FC (nFC), where improvements in optical and fluidic systems [such as adaptations with additional single-photon counting avalanche photodiodes (APD); Fig. 1(b)] have enabled more precise and specific analysis of EVs.^54^^,^^55^ nFC provides linear detection of particles ranging from 100 to 1000 nm in diameter covering both exosomes and larger EVs with multiplex fluorescent detection.^54^^,^^56^ nFC allows researchers to analyze multiple biomarkers on EVs, enabling comprehensive evaluation of EV cellular origins based on cell type specific markers, while gaining insight into disease pathology by labeling markers of disease.^54^^,^^57^^,^^58^

### 2.4 Label-Free Nonlinear Microscopy Approaches

Other label-free imaging approaches based on nonlinear microscopy strategies have recently been used to characterize EVs both in tissue microenvironments and in isolated samples. Similar to confocal reflectance imaging, which reveals aqueous medium-lipid membrane interfaces based on differences in refractive indices, third-harmonic generation (THG) microscopy uses differences in light scattering at these interfaces to efficiently reveal lipid membranes. You et al.^59^ took advantage of THG to capture diffraction-limited punctate signals of EVs, either in breast tissue excised from human females with invasive ductal carcinoma or individuals with no history of breast cancer, and then masked these EV signals using a deep neural network. The autofluorescence signal of the metabolic proteins FAD and NAD(P)H was subsequently quantified within the masked areas, enabling the researchers to use this as a measure of metabolic activity associated with the EVs, which was found to positively correlate with breast cancer diagnosis. Sun et al.^60^ integrated these principles into a multimodal intraoperative microscopy system for *ex vivo* imaging of freshly resected breast cancer tissues, combining two photon and three photon fluorescence with second and THG modalities. This multimodal setup enabled real-time imaging and quantification of EVs in unstained tissue and showed the spatial distribution of EVs alongside infiltrating tumor cells and dense collagen fibers, indicating that a higher number of EVs were present in the tumor microenvironment compared with healthy tissue (a finding which was verified by immunohistochemical labeling). Therefore, this label-free approach has been validated as a technique for in vivo imaging of EVs that could be incorporated into surgical procedures, making it a promising prognostic tool.

Raman spectroscopy and microscopy have also been used to evaluate EV content to characterize heterogenous EV subgroups in cancer samples. Raman shift profiles, generated based on the molecular vibration frequencies of specific molecules, can be used to evaluate the amino acid (tyrosine, phenylalanine, and tryptophan), fatty acid, nucleic acid (purines, pyrimidines, and imidazole rings), carotenoid, cholesterol, alpha-helix backbone, amide, lactic acid content. Raman spectroscopy is a label-free technique, so does not rely on antibody-based detection of biomolecules, and the sample is reusable after analysis.^61^ The signal in Raman spectroscopy may be enhanced with metal nanoparticles, leading to surface-enhanced Raman scattering (SERS), which has been used to successfully detect and characterize EVs in multiple myeloma patients during the progression of disease.^62^ However, while SERS may provide a few thousand potential SERS spectra for each EV sample, spatial variations in the distribution of enhancement factors limit the use of acquired spectra for quantitative analysis of heterogenous particles.^62^

Raman tweezers microspectroscopy (RTM) is a method which combines optical trapping and Raman probing for EV characterization.^63^ This technique has been used to characterize EVs released from different cancer cell lines^64^ and to differentiate EV subtypes in a number of studies.^65^^–^^67^ A significant advantage of RTM is its ability to obtain selective information from a single EV though the EV’s vibrational fingerprint, without the need for labeling.^68^ Its disadvantage is that the vibrational differences across EV subtypes may be difficult to detect. However, sensitive analysis methods were successfully implemented to identify vesicle subtypes and to demonstrate the differences between EVs from prostate cancer cells and EVs derived from healthy platelets and red blood cells.^67^

It should be noted that nonlinear optical imaging technologies require expensive and sophisticated hardware, particularly ultrafast pulsed lasers that may not be available in non-specialist laboratories.

### 2.5 Transmission Electron Microscopy

Transmission electron microscopy (TEM) is considered as the gold standard for visualization of single EVs. The spatial stability of electron beam and the chemical stability of the sample enables resolution lower than 1 nm since the electron wavelength is more than three orders of magnitude shorter than the visible light wavelength. Such high resolution allows the determination of size and morphology of EVs within a sample [Fig. 1(d)].^69^ However, the dehydration procedure during sample preparation and the vacuum environment required for TEM causes EV shrinkage, leading to underestimations of particle size and resulting in EVs with cup-shaped appearance.^70^^,^^71^ Furthermore, biochemical information about the composition of EVs may be obtained with immunogold labeling, where antibodies conjugated to gold nanoparticles are used to label specific biomolecules.^51^

### 2.6 Atomic Force Microscopy

Atomic force microscopy (AFM) allows topographical imaging at sub-nanometer resolutions. A cantilever with a sharp tip scans the surface of the sample without any physical contact and the movement of the tip is measured via a laser and photon detector to obtain a three-dimensional image without any prior staining and fixation [Fig. 1(e)].^72^^–^^74^ AFM has a 3-nm lateral and <0.1  nm vertical resolution, which makes it applicable for the size determination of EVs and it also outperforms DLS in the analysis of polydisperse samples.^35^ AFM can be used to measure the relative size distribution of EVs in their physiological state.^72^^,^^75^ Furthermore, by utilization of specific antibody-coated surfaces, EV subpopulations can be identified.^76^ Various AFM analyses modes have been used to characterize EVs, such as contact mode, tapping mode, non-contact mode, peak force tapping, and single molecule-force spectroscopy.^77^ However, the accuracy of AFM analysis is susceptible to experimental conditions, such as temperature, the state of the AFM tip, the force between the probe and sample, and variations in the scan speed.^61^^,^^72^^,^^78^

### 2.7 Western Blotting

Western blotting is commonly used to determine the presence of specific proteins in an EV preparation.^53^^,^^79^^–^^81^ Quantification and characterization of EV proteins provides insight into EV biology and can identify pathophysiological markers of the diseases.^53^ However, EVs are heterogeneous populations and there is no single protein or combination of proteins that is an universal EV marker.^82^ Accordingly, the International Society for Extracellular Vesicles recommends characterization of multiple transmembrane and cytosolic proteins enriched in EVs.^83^ In the set of guidelines proposed by the Society (Minimal Information for Studies of EVs; MISEV2018) for the isolation, characterization, and functional studies of EVs, it is recommended that at least one transmembrane protein (e.g., CD9, CD63, and CD81) and one cytosolic protein (e.g., TSG101, ALIX, and syntenin) is detected for positive identification of EVs in a preparation.^84^ Furthermore, quantification of common protein contaminants [e.g., apolipoproteins A1/2, albumin (ALB), and uromodulin (UMOD)] co-isolated with EVs from biofluids, such as plasma, urine, and culture medium, is recommended to evaluate the degree of EV purity.^84^

### 2.8 Enzyme-Linked Immunosorbent Assay

Enzyme-linked immunosorbent assay (ELISA) is another commonly used method for detection and quantification of EV proteins in a plate-based assay.^85^ In sandwich ELISAs, isolated EVs are applied to plates containing the capture antibody against the target EV antigen (e.g., CD63 and CD81), followed by detection of captured EVs using a second labeled antibody against a different epitope of the antigen, which increases the detection sensitivity and specificity. ELISAs are faster than Western blotting (Table 1), enabling high-throughput measurements.^53^^,^^85^ In general, sandwich ELISAs require low sample volumes and if sufficient sample volume is available the same sample may be applied several times for assessing different targets of interest.^85^ Although the levels of detected EV proteins may be used as a proxy measure for EV concentration, heterogeneous groups of EVs where EV marker proteins are not uniformly distributed limits any translations in protein concentration to EV subtype abundance.^79^^,^^85^^,^^86^ Additionally, ELISA is a multistep assay involving several washing steps, where potential problems, such as high background, poor replicate data, and weak signal can arise and affect the outcome. Moreover, intra-assay and inter-assay variability should be considered when planning experiment to maintain reproducibility between assays or to the consistency of sample replicates within an experiment. Hence, intra-assay and inter-assay variability could be a problem through technical errors.^85^

**Table 1:** Comparison of common EV characterization techniques.

Methods	Time required	Detection limit	Type of detection	Advantages	Disadvantages
DLS	<1 h	1 nm to 6 μm	Size of particles	• Determines sizes of monodisperse samples over a large size range • Analysis of particles in solution	• Requires specialist equipment • Only provides bulk analysis of heterogeneous EV populations • Accurate size measurements limited to monodisperse samples • Unable to distinguish EVs from protein contaminants
NTA	<1 h	60 to 1000 nm	Size distribution and concentration of particles	• Limited sample preparation required • Detects relatively low concentrations of particles • Analysis of particles in solution • Option for fluorescence detection of EV marker proteins to increase specificity	• Requires specialist equipment • Bias toward detection of larger particles • Difficulties in distinguishing EVs from other particulates • Limited size detection
FC	2 to 3 h	300 to 600 nm	Fluorophore-conjugated antibodies bound to extravesicular proteins	• High-throughput • Multiple channels available to detect several proteins in the same sample • Low amount of sample required	• Only specialist flow cytometers are able to detect single EVs (<300 nm) • Standard FC is unable to distinguish individual EVs from aggregates • Bead-based detection of EVs only provides a bulk measurement of heterogenous samples
TEM	2 to 3 h	0.5-nm microns	Morphology and sizes of individual EVs	• Direct imaging of EVs • Option to label EVs with gold-conjugated antibodies to detect specific proteins	• Imaging EVs in their non-native state • Underestimates EV size due to shrinkage caused by the dehydration procedure • Low throughput
AFM	Several hours	0.5-nm several microns	Morphology, mechanical properties, and size distribution of EVs	• Characterization of multiple parameters in polydisperse samples • Option to image in liquid to analyze EVs in their native state	• Low throughput • High equipment cost • Requires immobilization of EVs on a substrate
Western blotting	Several hours	NA	Fluorophore/enzyme-conjugated antibodies bound to extravesicular proteins	• Provides semi-quantitative detection of specific EV proteins	• Unable to detect low abundance proteins in a sample • Bulk analysis technique unable to determine protein heterogeneity in EV populations within a sample
ELISA	90 min to several hours	NA	Fluorophore/enzyme-conjugated antibodies bound to extravesicular proteins	• Provides semi-quantitative detection of proteins in an EV preparation • Multiwell analysis enables high-throughput analysis	• Unable to detect heterogeneity in an EV preparation • Unable to distinguish EVs from other biological material because of a lack of EV-specific markers
Confocal microscopy	<1 h	200 to 300 nm	Fluorophore-conjugated antibodies bound to extravesicular proteins and reflectance signals from vesicle membranes	• High-throughput • Rapid, direct imaging of EVs • Low amount of sample required • Enables detection of multiple EV subtypes in the same sample, so can be used for fluorescent multiplexing • Combines reflectance and fluorescence modalities to distinguish proteins in EVs from free protein	• Diffraction limit prevents identification of single EVs and differentiating them from an EV cluster • Requires fixation and immobilization of EVs on a surface • True size of EVs cannot be measured

### 2.9 Optical Microscopy

The maximum diffraction-limited resolution that can be obtained with optical microscopy is around 200 to 300 nm. Therefore, it is practically impossible to visualize the fine structure of small EVs like exosomes and distinguish them individually when they are very close to each other or inside aggregates without using sophisticated techniques. Nevertheless, it is still possible to detect fluorescent emission or other optical signals, such as scattered light from single EVs if they are sparsely dispersed in the preparation, which can be valuable for their phenotypic characterization. Direct fluorescent labeling of EVs can be achieved using lipophilic dyes, such as DiI, DiD, or DiC that fluoresce when bound to lipid membranes.^87^ Nucleic acid indicators such as acridine orange or thioflavin T can also indicate the presence of EVs by binding to vesicular RNA.^88^ However, scattering signals or lipophilic fluorescence is of limited use for phenotypic characterization and differentiation of EV populations. For this purpose, EVs must be tagged with fluorophore-conjugated antibodies against their specific proteins (i.e., immunofluorescent labeling) that can indicate their origin when targeting proteins specific to EVs of different cell types.^37^ Alternatively, the EV parent cell may be genetically modified to express fluorescent reporters on proteins that are then incorporated into EVs specifically released by that cell.^89^^,^^90^ To enhance diffraction-limited fluorescent signals comparable to EV size, nanobodies (fragments of antibodies from camelids or sharks^91^) can be utilized. These have a smaller size (15 to 25 kDa) than whole immunoglobulins (∼150  kDa) and can be conjugated to a variety of organic fluorophores or quantum dots.^92^ Quantum dots can provide brighter fluorescence signals with a more stable and narrow-band emission compared with conventional fluorophore-conjugated antibodies or transgenically expressed fluorescent reporter proteins on EVs,^93^ and they are suitable for surface modifications and labeling with specific molecules of interest.^94^^–^^96^

Unless EVs are captured on a special surface or device that will separate individual EVs in a monolayer, wide-field epifluorescent illumination will be of limited value because the whole specimen will be illuminated and precise signal localization of the EVs will not be achievable. Therefore, high-resolution imaging techniques such as total internal reflectance microscopy, confocal microscopy, or novel tools like stimulated emission depletion (STED) are necessary to increase the resolution to the optical diffraction limits and to limit signals to those originating from the focal plane of the EVs.^87^^,^^92^ Despite challenges in fixing EVs on a glass slide and focusing on the extremely thin EV layer, it has been shown that confocal microscopy could visualize EVs immunolabeled with antibodies.^41^ Even multiplexed profiling of single EVs is possible by combining fluorescence microscopy with microfluidic immobilization techniques.^97^^,^^98^ STED microscopy allows far better spatial resolution. For instance, a diameter as small as 16 nm can be imaged, which provides sufficient resolution for EV characterization.^41^^,^^99^^,^^100^ In addition to determination of size and localization of the fluorescently labeled EVs, STED can also be used for morphological characterization of EVs, as well as examining the distribution of the labeled proteins on the EV surface.^101^^,^^102^ Direct stochastic optical reconstruction microscopy (dSTORM) has also been employed to characterize EVs [Fig. 1(c)],^41^ but this technique is limited to fluorophores with photoactivation and blinking properties.

There are also several limitations associated with fluorescent labeling that need to be considered. For instance, antibodies forming aggregates or binding to Fc receptors rather than the target antigens on EVs might give non-specific signals.^103^ Moreover, autofluorescence or irreversible photobleaching of the fluorophore can complicate the detection of a specific fluorescence signal.^104^ In a biological sample, there are numerous sources of nonspecific particulate-like signals that can be misinterpreted as EV-related signals. Elimination of these signals is essential and can be achieved by adding a second imaging modality, such as scattering or reflection. EVs scatter and reflect light from their lipid membranes, which provides different refractive indices compared to the surrounding aqueous medium.^51^^,^^87^ A pinhole in front of the detector is essential for this imaging to eliminate the out-of-focus light and to increase the minor contrast of reflected light intensity.

### 2.10 Combined Reflectance and Fluorescence Confocal Microscopy of EVs

Recent work in our laboratory has shown that confocal reflectance microscopy can be sufficient for identification of EVs with high signal-to-noise ratio and can be used to differentiate the specific fluorescence signals originating from EVs from artifactual signals. We have been able to visualize and phenotypically characterize EV samples from mouse brains by simultaneously using reflectance and fluorescence modes of a confocal laser scanning microscope; a method that does not require additional specific or high-cost equipment. Therefore, our approach detailed below, can be implemented in a laboratory with a confocal microscope and can allow rapid and low-cost characterization of the EVs in a biological sample, providing a practical method for EV profiling.

**Fig. 2:**
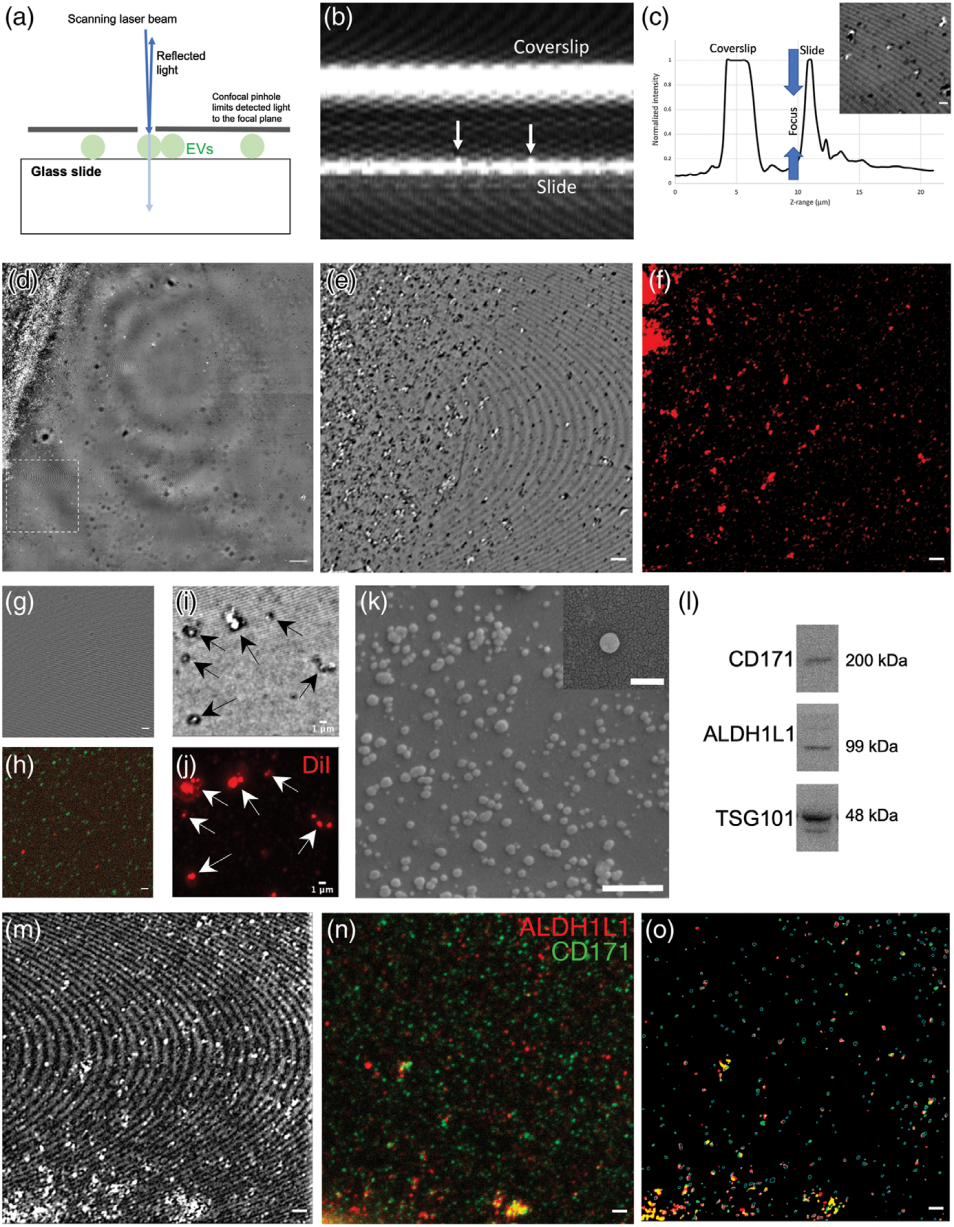
(a) Diagram demonstrating the reflectance signals that originate from the laser-scanning of EVs fixed on a glass slide. (b) Orthogonal x−z plane image of a Z-stack of reflectance images indicating the highly reflective coverslip and surface of the glass slide; these are cues that can aid in finding the focal plane of EVs. Arrows indicate punctate reflectance signals from EVs on top of the glass slide. (c) Normalized signal profile of the reflectance signals, illustrating the peaks of highly reflective glass surfaces. Imaging focus was adjusted to slightly above the upper surface of the slide (d) Wide-field confocal reflectance image of the EV sample shows the thick aggregate that forms at the boundary of the drop and smaller aggregates closer to the center. Dotted square indicates the imaging field in (e) and (f). Scalebar: 10  μm. (e) Close-up view of the boxed zone in (d) shows bright reflection signals from EVs and their clusters. Scale bars: 2  μm (f) immunolabeled EVs for L1CAM indicate their neuronal origin. (g) and (h) PBS control had no reflection signals but had punctate nonspecific fluorescence signals suggesting free unbound antibody complexes or autofluorescent elements. Scale bars: 2  μm. (i) and (j) Staining of the high-intensity reflectance particles with DiI (a lipophilic dye), indicating that they are membranous particles. Scale bars: 1  μm. (k) SEM images of the EVs derived from mouse brain cortices (scale bar: 500 nm), and a high magnification image of a single EV from the same sample (inset, scale bar: 200 nm). (l) Representative immunoblot images of L1CAM (CD171), ALDH1L1, and TSG101 proteins in brain-derived EV sample. (m)–(o) Reflection signals were used to select fluorescence signals originating from reflection-positive EVs and to mask all other nonspecific signals. It is noteworthy that not all EVs are labeled with either ALDH1L1 or L1CAM, suggesting that they are not of astrocytic or neuronal origin. Scale bars: 2  μm.

We used reflectance signals in focusing because sharp focusing of the EVs in fluorescence mode was difficult due to their small size. When acquiring Z-stacks in the reflectance mode, the coverslip and the glass slide created two high-intensity reflection planes. We used these peak signals as references to set the focus to slightly above the bottom of the glass slide where the EVs were located [Figs. 2(a)–2(c)]. Four independent researchers, after being given an initial introduction to the technique, successfully identified the EV focal plane with guidance from reflectance signals, showing the inter-operator reliability. Filtered PBS was used as control for nonspecific reflectance and fluorescence signals [Figs. 2(g) and 2(h), respectively]. Our setup provided a lateral resolution of 198 nm and axial resolution of 492 nm for 488 nm reflected light imaging. Microscopic areas of $40\times 40\;{\mu m}^{2}$ were imaged with 1024×1024  pixel scanning. Z-stacks of 1.2  μm were acquired with 0.3-μm steps. Maximum intensity projections were used for visualization purposes and analyses. EVs and their aggregates had small and bright reflection signals compared to the background, which allowed for their identification [Figs. 2(d), 2(e), and 2(k)]. When the confocal microscope was in fluorescence mode, EV fluorescence was clouded by numerous nonspecific punctate fluorescence signals, but aligning reflectance images with the fluorescence images enabled discrimination of EV fluorescence from nonspecific signals. It should be noted that nonspecific fluorescence without reflectance was observed even in PBS samples [Figs. 2(g) and 2(h)]. However, in the corresponding reflectance image [Fig. 2(g)], there was no matching reflectance signal which shows that these particles were not EVs; thus, providing the ability to distinguish between the fluorescence signal from EVs and non-specific fluorescence signal. Therefore, adding the reflectance modality clearly helped in selecting the specific fluorescence signals originating from membrane-bound EVs. We confirmed that the reflectance positive signals were membrane-bound vesicles by staining with DiI, a lipophilic dye [Figs. 2(i) and 2(j)].

We also noticed that applying the EVs onto the glass slides in drops led to the accumulation of EVs at the boundaries of the drop where they formed aggregates [Fig. 2(d)]. These aggregates were not observed in PBS controls. This occurred even though the drops containing the EVs were not allowed to dry out, probably because of physical particle interaction dynamics at the nanoscale.^105^ Therefore, the central zones of drops were mostly devoid of EVs, whereas thick EV aggregates were formed at the periphery. To visualize smaller EV clusters with less aggregation, we imaged the zone immediately adjacent to the thick EV aggregate (closer to the center of the original droplet) [Fig. 2(e)].

By calculating the fraction of positive pixels for both reflectance and fluorescence in each image, we could quantify the density of EV particles and the percentage of EVs expressing fluorescence signals of particular interest (e.g., L1CAM-positive). In our samples, 40.4±6.1% and 44.8±4.5% of EVs were positive for neuronal L1CAM and astrocytic ALDH1L1, respectively. Western blot imaging was also performed for further characterization of brain-derived EVs to compare our proposed technique with the existing methods. Immunoblot images revealed that EVs of neuronal and astrocytic origin are both significantly present in our sample in line with microscopy findings [Fig. 2(l)]. Intriguingly, although there should be no vesicle expressing both markers from the two different cellular populations, we observed a number of fluorescence signal overlaps between L1CAM and ALDH1L1; 18.7±10.3% of EVs were positive for both markers. This indicates that a fraction of the particulate signals from EVs correspond to an aggregation of vesicles of different cellular sources which could not be resolved individually because of the optical resolution limits of confocal microscopy. It should also be considered that, because this technique depends on scattered and reflected light (similar to NTA), it may be biased to the detection of larger EVs.^106^ While we were not able to perform sensitivity analyses because of technical limitations, we performed scanning electron microscopy (SEM) to verify the vesicular contents of the sample. This showed the presence of smaller vesicles (<150  nm) and some larger aggregates [Fig. 2(k)] which could account for the potential aggregate signal observed by confocal microscopy.

The proof-of-concept data presented here suggest that the integrated use of reflectance and fluorescence confocal microscopy enables rapid and low-cost screening of EV samples isolated from the brain. This will be useful for practical purposes such as evaluating the isolation efficiency of EV subpopulations bearing certain proteins, screening for the effects of physiological changes on the abundance of specific proteins, and to indicate colocalization of particular proteins on EVs, before proceeding with more sophisticated and confirmatory EV quantification and analysis tools. It should be noted that our approach will need to be further evaluated in future studies to determine its practical utility and the limitations of the methodology which we have introduced here.

## 3 Conclusion

The analysis of brain-derived EVs has already increased our understanding of the mechanisms of neuropathologies. For example, brain endothelium-derived EVs isolated from the plasma of patients with small cerebrovascular disease were found to carry elevated levels of complement mediators compared with controls, indicative of an inflammatory phenotype in brain endothelial cells which could contribute to cerebral white matter injury.^107^ However, despite these advances, the small size and heterogeneous nature of EVs mean that their characterization remains a challenging task. Classical techniques such as Western blotting and ELISA can be helpful for evaluation of their content and surface markers, and electron microscopy is still the gold-standard for their morphologic evaluation, but these approaches either require large amounts of sample, lengthy procedures, specialist equipment, or do not provide information about the molecular heterogeneity in an EV preparation. Advances in optical strategies, like improving tools such as NTA or direct microscopic imaging, may improve EV detection and phenotypic characterization in a high-throughput manner, which will help elucidation of their biological and pathological value in different experimental environments and clinical samples (Table 1). Here, we present a technique combining the reflectance and fluorescence modes of a confocal microscope (available in many cell biology laboratories) to characterize molecularly distinct subpopulations in EV preparations which could be incorporated into the already available framework for EV characterization as a complementary approach without the need for specialist equipment. As single-EV technologies emerge as key tools to overcome earlier limitations in analyses of EV heterogeneity (as recently reviewed by Ref. 108) this combined confocal and reflectance microscopy technique could be a useful initial analytical step to indicate the EV diversity in a preparation before performing more time and resource intensive single-EV analyses.

## 4 Methods

### 4.1 Brain-Derived Extracellular Vesicle Enrichment

EVs were isolated from the brain cortex of 8- to 12-week-old male Swiss albino mice (n=4) using a commercially available EV isolation kit (Thermo Fisher Scientific, Waltham, Massachusetts, United States; Catalog No: 4484450) according to manufacturer’s instructions. Surgical procedures were carried out under chloral hydrate anesthesia (1000  mg/kg, intraperitoneal) in accordance with the institutional guidelines and as approved by the Hacettepe University Animal Experiments Local Ethics Committee (2021/50). After decapitation, the cortex was removed, placed on ice and 500  μL of ice-cold phosphate-buffered saline (PBS) was added on the sample. The tissue was homogenized using a MACS homogenizer and the homogenate was further washed with 500-μL PBS. Next, it was centrifuged at 1500×g for 10 min at 4°C and the supernatant was transferred to another tube, vortexed, then centrifuged again at 10,000×g for 30 min at 4°C. The supernatant was then transferred to a new tube, vortexed again and the exosome extraction reagent from the EV isolation kit was added to the sample (0.2×sample volume) before incubating at room temperature (22°C to 23°C) for 10 min. Then, the sample was centrifuged at 10,000×g for 10 min at room temperature and the obtained EV-enriched pellet was dissolved in 200-μL autoclaved and 0.22-μm filtered PBS, which was also used for the preparation of diluent and washing steps during immunofluorescent labeling.

### 4.2 Immunofluorescent Labeling

Poly-l-lysine-coated glass slides and coverslips were cleaned with ethanol, which was allowed to completely evaporate before use. About 10-μL of EV sample was mixed with 10-μL 4% PFA (giving a 2% final PFA concentration), then immediately pipetted onto a slide in various small droplets of 2  μL. Next, the slides were incubated at room temperature in a humidified chamber for 20 min to allow the EVs to adhere to the slide surface. EVs were then immunolabeled with antibodies against ALDH1L1 and L1CAM to determine the cell type from which the EVs were released. Briefly, either a mouse monoclonal L1CAM antibody (Abcam ab24345, 1:200 dilution) or rabbit polyclonal ALDH1L1 antibody (Abcam ab87117, 1:200 dilution) were used followed by secondary labeling with goat anti-mouse IgG-Alexa Fluor 594 (Abcam ab150116, 1:200 dilution) and goat anti-rabbit IgG-Alexa Fluor 488 (Abcam ab150077, 1:200 dilution). Incubations with both primary and secondary antibodies were carried out at room temperature for 1 h. Antibody diluent was 1% bovine serum ALB (BSA) in 1× PBS. The samples were mounted in autoclaved and 0.22-μm filtered PBS.

### 4.3 Confocal Microscopy

A laser scanning confocal microscope (Leica SP8) and an oil immersion objective (63×, NA: 1.40) were used for imaging. The image acquisition was obtained in both reflectance and fluorescence modes switched from one to another using the LASX acquisition software. For reflectance imaging, excitation was done using the 488 nm visible light laser, with the RT 15/85 beamsplitter. Spectral PMT detection range was adjusted to 470 to 500 nm. For fluorescence imaging, excitation was done at 488 nm for Alexa488 and 552 nm for Alexa 594 fluorophores, using the double dichroic 488/552 beamsplitter. Reflectance signals were used for focusing. When acquiring Z-stacks in the reflectance mode, the coverslip and the glass slide created two high-intensity reflection planes. Filtered PBS was used as control for nonspecific signals. Our setup provided a lateral resolution of 198 nm and axial resolution of 492 nm for 488 nm reflected light imaging. Microscopic areas of $40\times 40\;{\mu m}^{2}$ were imaged with 1024×1024  pixel scanning. Z-stacks of 1.2  μm were acquired with 0.3-μm steps.

### 4.4 Image Analysis

All image-processing steps were performed using FIJI/ImageJ (Version 2.1.0/1.53f). We first registered reflection images to the fluorescence images, using manual landmarks. A bandpass filter of 3 and 30 pixels were applied along with background subtraction with a sliding window of 30 pixels in both reflection and fluorescence images to improve signal-to-background ratio. Then, an intensity-based threshold was applied in reflectance images to select the top 3% of pixels, to form binary masks of EV signals. This mask was applied over fluorescence images to evaluate signals coming only from reflection-positive particles [Figs. 2(l) and 2(m)]. A threshold was applied to fluorescence images with the default algorithm of FIJI/ImageJ and were converted to binary images. This approach allowed us to classify pixels corresponding to either the reflectance or fluorescent signals or both.

### 4.5 Western Blotting

Following determination of the protein concentration using the Pierce BCA protein assay kit (Thermo Fisher Scientific, 23225), 35-μg protein/well were run on NuPAGE 4% to 12% Bis-Tris Protein Gels (Thermo Fisher Scientific, NP0321BOX), then transferred to PVDF membranes. After transfer, membranes were blocked in TBS containing 0.5% tween-20 and 5% BSA for 1 h at room temperature. Next, membranes were incubated overnight at 4°C with primary L1CAM (Abcam ab24345, 1:1000), ALDH1L1 (Abcam ab177463, 1:1000), and TSG101 (Abcam, 1:500) antibodies diluted in blocking solution, then with secondary goat anti-mouse (Abcam ab6789, 1:5000) or goat anti-rabbit (Abcam ab6721, 1:5000) HRP conjugated IgG antibodies for 1 h at room temperature. Band detection was performed using SuperSignal West Femto Maximum Sensitivity Substrate Kit (Thermo Fisher Scientific, 34095). Densitometric band analyses were performed with ImageJ software.

### 4.6 Scanning Electron Microscopy

The EV sample preparation was diluted 1:10,000 in ${\mathrm{ddH}}_{2}O$, then 10  μL was left to dry at room temperature on a slide attached to a stub with a carbon tape before being coated with 4 nm gold palladium. SEM images were acquired using a TESCAN-GAIA3 FIB-SEM operated at 4 to 5 kV and scan speed 6–7.
